# Medical Life in Philadelphia

**Published:** 1852-11

**Authors:** 


*Medical life in Philadelphia.* — In the following extract from the Philadelphia Medical and Surgical Journal, Dr. Bryan, the editor, gives a sorry picture of the condition of medical matters in Philadelphia. There is some comfort in the reflection that in most parts of the country the practice of medicine is in better estimation with the public:

"Two-thirds of the practice of medicine in Philadelphia, even among those who pay for everything else, is done gratis; and not unfrequently insult is added to injury. This is due to the crowded state of the profession, and to the *mock charity* inculcated by many of the public teachers. The average fee in our city for medical attendance *paid*, does not exceed *twenty-five cents* a visit. This is one great reason that quackery so thrives among us. Perhaps there is not a city in the Union where quackery, when, as it often is, is combined with talent, builds such large houses (vide the palace to Moloch, on Chestnut street,) as among the staid and demure denizens of the City of Brotherly Love. The getting of fees is a kind of game in which the honorable and upright are fleeced to pay for the mean and contemptible. *Vive la bagatelle.*"

